# Characterization of Receptor Binding Affinity for Vascular Endothelial Growth Factor with Interferometric Imaging Sensor

**DOI:** 10.3390/bios14070315

**Published:** 2024-06-24

**Authors:** Nese Lortlar Ünlü, Monireh Bakhshpour-Yucel, Elisa Chiodi, Sinem Diken-Gür, Sinan Emre, M. Selim Ünlü

**Affiliations:** 1 Faculty of Medicine, Histology and Embryology, Atlas University, 34408 İstanbul, Turkey; 2 Photonics Center, Department of Biomedical Engineering, Boston University, Boston, MA 02215, USA; selim@bu.edu; 3 Department of Chemistry, Faculty of Arts and Science, Bursa Uludag University, 16059 Bursa, Turkey; myucel@uludag.edu.tr; 4 Photonics Center, Department of Electrical Engineering, Boston University, Boston, MA 02215, USA; elich@bu.edu (E.C.); sinemmdiken@gmail.com (S.D.-G.); 5 Department of Biology, Hacettepe University, 06800 Ankara, Turkey; 6 Batigoz Eye Health Branch Center, 35210 Izmir, Turkey; mdsinanemre@yahoo.com

**Keywords:** Age-related macular degeneration (AMD), Vascular Endothelial Growth Factor (VEGF), anti-VEGF drug, aflibercept, bevacizumab, Interferometric Reflectance Imaging Sensor (IRIS)

## Abstract

Wet Age-related macular degeneration (AMD) is the leading cause of vision loss in industrialized nations, often resulting in blindness. Biologics, therapeutic agents derived from biological sources, have been effective in AMD, albeit at a high cost. Due to the high cost of AMD treatment, it is critical to determine the binding affinity of biologics to ensure their efficacy and make quantitative comparisons between different drugs. This study evaluates the in vitro VEGF binding affinity of two drugs used for treating wet AMD, monoclonal antibody-based bevacizumab and fusion protein-based aflibercept, performing quantitative binding measurements on an Interferometric Reflectance Imaging Sensor (IRIS) system. Both biologics can inhibit Vascular Endothelial Growth Factor (VEGF). For comparison, the therapeutic molecules were immobilized on to the same support in a microarray format, and their real-time binding interactions with recombinant human VEGF (rhVEGF) were measured using an IRIS. The results indicated that aflibercept exhibited a higher binding affinity to VEGF than bevacizumab, consistent with previous studies using ELISA and SPR. The IRIS system’s innovative and cost-effective features, such as silicon-based semiconductor chips for enhanced signal detection and multiplexed analysis capability, offer new prospects in sensor technologies. These attributes make IRISs a promising tool for future applications in the development of therapeutic agents, specifically biologics.

## 1. Introduction

Age-related diseases reduce the life quality of the elderly population, hampering the execution of the simplest life tasks, leading in some cases to early hospitalization, depression and social isolation [1]. Gradual vision loss is one of the most common conditions that affect the geriatric population in industrialized countries, and the main causes of vision loss can be mentioned as chronic eye diseases, like cataracts, glaucoma and macular degeneration. While glaucoma principally affects peripheral vision, impacting balance and motor skills such as walking and driving, cataracts and macular degeneration mostly impact central vision, therefore causing an impairment in performing daily activities such as reading, distinguishing faces and using screen-based devices such as cellphones or computers [2].

Age-related macular degeneration (AMD) is nowadays the leading cause of blindness in the world population aged 50 and older. The progressive, chronic disease can occur in two forms, neovascular (wet) and atrophic (dry); the latter is caused by atrophic spots in the macula, which is the central part of the retina located in the back of the eye, responsible for detailed vision [1]. Neovascular—or wet—AMD is caused by the abnormal growth of blood vessels (angiogenesis) in the same retinal region, due to the excessive expression of Vascular Endothelial Growth Factor (VEGF) [3]. Old age is the major factor in the emergence of the disease; however, some systemic risk factors have also been identified, such as smoking, obesity, a low intake of nutrients such as vitamins and zinc and an unhealthy lifestyle that correlates with cardiovascular diseases [1]. Increased VGEF levels have been determined in the retinal vasculature and vitreous fluid of patients diagnosed with AMD. A promising therapeutic approach for both AMD and other ocular diseases caused by abnormal angiogenesis is based on treatment with anti-VEGF antibodies to block VEGF activity [4,5].

Alternative drugs have been developed and compared with each other to find the most effective drug that can be used with a lower dose and less frequently, such as VEGF trap-aflibercept (Eylea, Regeneron) and bevacizumab (Avastin, Genentech). Aflibercept acting as an inhibitor of both VEGF and PlGF (Placental Growth Factor) is a recombinant (fusion) protein component of VEGF receptors (VEGF 1 and VEGF 2). Each aflibercept molecule has multiple binding sites for both VEGF-a and VEGF-b, yielding a much higher (100×) binding affinity for the target with respect to commercially used ranibizumab [6]. This results in a more long-lasting effect per dose, allowing for less frequent administration and therefore a lower overall cost, benefiting lower-income patients. On the other hand, bevacizumab, a monoclonal antibody, was initially developed as a treatment for colon cancer, and its cost is also high. However, it is sometimes used as an ‘off-label’ solution for AMD patients; since only a fraction of a dose is needed, the cost is reduced [7].

In studies over the last decade, comparing the VEGF binding capacity of developed drugs, sensor systems such as surface plasmon resonance (SPR) have been preferred instead of Enzyme-linked immunosorbent assay (ELISA) due to its real-time detection capability [4,8,9,10]. However, environmental factors such as temperature changes, pH and solvent variations that caused changes in the refractive index of the solution inevitably raise the noise of SPR measurements. Moreover, multiplexed measurement cannot be performed with traditional SPR, and surface plasmon resonance imaging (SPRi) can be used to achieve multiplexed detection instead. However, the main challenge that limits SPRi usage in pharmacology research is the inability to measure the binding kinetics of small molecules such as drugs as the optical response formed by the accumulation of these molecules is below the detection limit of the method. Additionally, the high cost of the consumables needed for SPR analysis, especially the chips which are manufactured by valuable metals such as gold or silver, is an important issue that restricts their widespread use in the clinic [9,11].

In this study, we show that our interferometric sensing method enables the quantitative comparison of the binding affinity of the drugs to their ligands and demonstrates a specific capability on biologic drugs by measuring the binding affinity of aflibercept and bevacizumab to their target VEGF. One of the key advances that distinguishes IRISs from other sensor systems is the use of a low-cost Si-based chip as a multi-sensing platform. Thanks to the well-established semiconductor manufacturing industry, Si-based chips of an IRIS platform can be produced in large amounts, and the cost of each microfluidic cartridge is less than USD 5 [9,12]. On the other hand, many biomedical companies are selling SPR chips at a price range of USD 200–1000 per chip [13,14,15]. Therefore, the versatile features of IRIS technology make it a good candidate for the measurements of the binding affinity of VEGF to anti-VEGF therapeutics.

## 2. Materials and Methods

### 2.1. Materials

Aflibercept (Eylea) was purchased from Regeneron, Inc., Tarrytown, NY, USA, and bevacizumab (Avastin) was obtained from Genentech, Inc., San Francisco, CA, USA. Recombinant human VEGF165 protein expressed in HEK293 cells was purchased from SinoBiological, Inc., Wayne, PA, USA. The lyophilized recombinant human VEGF (rhVEGF) was dissolved in filtered NanoPure water, then aliquoted and stored at −20 °C until use. Human immunoglobulin G (IgG) and Bovine serum albumin (BSA) were obtained from Sigma Aldrich, Saint Louis, MO, USA. 1X phosphate-buffered saline (PBS) was purchased from Lonza, Inc., Basel, Switzerland. IRIS chips were purchased from Silicon Valley Microelectronics, Santa Clara, CA, USA. N, N-dimethylacrylamide (DMA)-based polymer MCP-2 utilized for the coating of the chips’ surface was generously provided by Dr. Marcella Chiari.

### 2.2. Interferometric Reflectance Imaging Sensor

**Figure 1:**
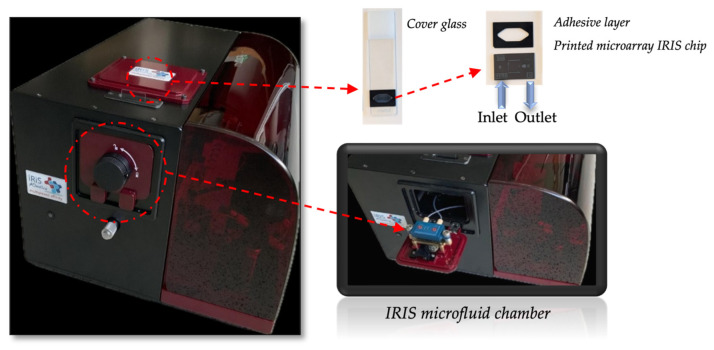
Scheme of prototype IRIS instrument.

An IRIS is a reflectance-based sensor which allows us to monitor biomass accumulation on top of a layered substrate. The device was developed by our group (shown in Figure 1), and its working principle has been thoroughly described in the literature [16,17]. Briefly, a silicon chip coated with a thin layer of silicon dioxide (110 nm of SiO_2_) is illuminated from the top, through the solution, with a 4-color LED system with wavelengths of 457, 518, 595 and 632 nm, through a 2× objective. The reflected light is collected through the same objective and then directed by a beam splitter towards a CCD camera. The thickness of the oxide layer on the chip is engineered such as to provide high sensitivity to surface binding (biomass accumulation) while simultaneously providing insensitivity to background solution variations (bulk effect). Reflection images are recorded in real time (with the current configurations temporal limit at 10 ms) as analytes are captured (biomass accumulates) on designated areas on the sensor chip corresponding to an array of capture probes (ligands). Image analysis produces multiple binding curves by calculating the change in the measured reflectance signal.

### 2.3. Preparation of Chip

To prepare the microfluidic cartridge, holes were first drilled with a laser at both ends of the Si/SiO_2_ substrate of the IRIS chip that allow liquids to enter the chamber, flow through the chip and exit. Then, the silicon chip surface was functionalized by coating with an active polymer (MCP-2) and spotted with anti-VEGF drugs to create a microarray on the chip surface. As a control, BSA and IgG were also spotted on the chip surface.

Finally, anti-reflection (AR)-coated glass was placed on the chip surface through an adhesive silicon spacer with a thickness of 130 μm. AR coating was utilized to prevent unwanted reflection caused from the air–glass interface. Before functionalization with MCP-2, the chip surface was activated by using oxygen plasma for 10 min. Afterwards, the activated chip was immersed into the polymer solution (1% w/v MCP-2 dissolved in 20% saturated ammonium sulfate) for 30 min. Then, the coated chip was washed with distilled water and first dried with N_2_ and then dried by a vacuum oven at 80 °C for 15 min. Anti-VEGF drugs, aflibercept and bevacizumab, and control spots (BSA and IgG) were ultimately spotted on the surface of the chip in a microarray modality using an M2 iTWO spotter, and then the chip was kept overnight at 70% humidity. Prior to the assay, the surface of the chip was blocked with 100 mM Tris and 50 mM Ethanolamine (pH 9) solution to prevent the non-specific binding of the analyte to the chip surface, then washed with distilled water and dried under N_2_. The characterization of the bare chip and MCP-2-coated IRIS chip was realized using water contact angle (Attention KSV Instrument, Espoo, Finland), optical profilometer (Zeta Instruments, San Jose, CA, USA) and Ellipsometry (Nanofilm EP3, Goettingen, Germany) instruments. The contact angles of the bare chip and the MCP-2-coated chip surface were obtained. Various regions of the chip surface were selected, and photographs were captured to measure the contact angles.

To ascertain the thickness of the MCP-2 polymer coating on the IRIS chip surface, optical profilometer measurements were conducted at four different regions.

### 2.4. IRIS Binding Assays

The binding affinities of anti-VEGF drugs to rhVEGF were evaluated by using IRIS technology. Firstly, rhVEGF was prepared in phosphate buffer, pH 7.4 at a final concentration of 1 nM, and injected to the chamber with a flow rate of 50 µL/min to provide the flowing of solution along the surface of the chip. After the injection of rhVEGF, changes occurred in the reflectance signal due to the biomass accumulation on the aflibercept and bevacizumab spotted area (IRIS-A and IRIS-B) of the chip that were measured simultaneously owing to the multiplexed imaging capacity of IRISs. Improving the signal levels obtained by the binding interaction between anti-VEGF drugs and their target rhVEGF, the sandwich assay model was applied as a second experiment. In this model, bevacizumab and aflibercept molecules were injected into the chamber to detect changes in signal levels after binding to rhVEGF, which was bound to the spotted drugs on the chip. Lastly, to examine the signal level difference obtained with the sandwich assay model on the same diagram, bevacizumab and aflibercept molecules were given to the chamber immediately after the injection of rhVEGF. Triplicate experiments were performed to obtain accurate results.

### 2.5. Image Analysis

Images of the entire field-of-view of the sensor chip were acquired during the experiments. When evaluating the images acquired in real time, two different regions were considered for each spot: spot point (Sspot) and a round-shaped region around the spot point that was defined as the background (Sbg). The binding curves were procured according to the equation ΔS = Sspot − Sbg that shows the differential change in the signal. Then, the data that depended on time were recorded and analyzed with software on the instrument (detection of binding between anti-VEGF drugs and rhVEGF), and more sophisticated analysis was performed using MATLAB codes offline.

## 3. Results and Discussion

Recently, the limitations of the detection methods used today to investigate biomolecular interactions have prompted innovative research groups to develop new sensor systems that offer the opportunity for label-free, cost-effective, high-sensitivity and rapid sensing. In this context, an IRIS that possesses the above-mentioned benefits is a qualified method for investigating the binding affinity and kinetics of biomolecules, also in studies such as drug development and the determination of therapeutic efficacy. Moreover, in the past few years, IRIS technology has proven its success for several biomedical applications including clinically important bacteria and virus detection, binding affinity investigations of low-molecular weight drugs and toxins and the detection of diagnostic biomarkers by providing the multiplexed detection of binding events simultaneously [9,18,19,20].

The investigation of the binding affinity of a novel therapeutic drug to its target constitutes the most crucial part of pharmaceutical developments. In the present study, primary molecular binding interactions between rhVEGF and its inhibitors aflibercept and bevacizumab were directly monitored with a low-cost IRIS cartridge to evaluate the effectiveness of the IRIS technique in the field of pharmaceutical research. While examining the binding affinity of anti-VEGF drugs to their target, the main challenges for obtaining monovalent binding affinity for rhVEGF and its inhibitors are the dimeric form of rhVEGF and the presence of two binding sites in the inhibitors [7]. A low density of the analyte (rhVEGF, 1 nM) flowed across the bevacizumab and aflibercept spotted chip in order to promote monovalent binding.

**Figure 2:**
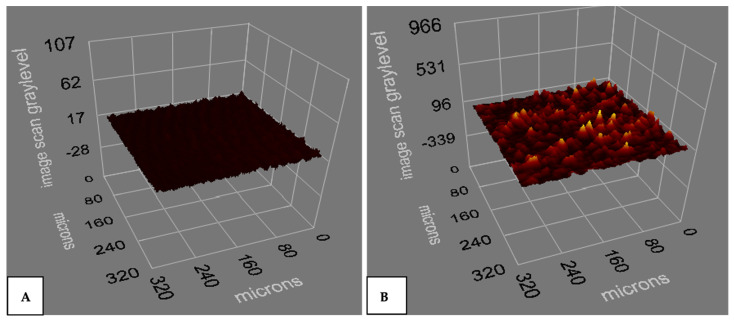
The thickness of the (**A**) bare Si-SiO_2_ chip and (**B**) functionalized Si-SiO_2_ chip obtained by ellipsometry.

Firstly, functionalized and bare Si-SiO_2_ IRIS chips were characterized by ellipsometry, WCA and optical profilometer measurements to investigate the effect of the functionalization technique. According to the ellipsometry results, the thickness of the oxide and MCP 2 polymer coating were found as 17 and 96 nm, respectively (Figure 2A,B).

**Figure 3:**
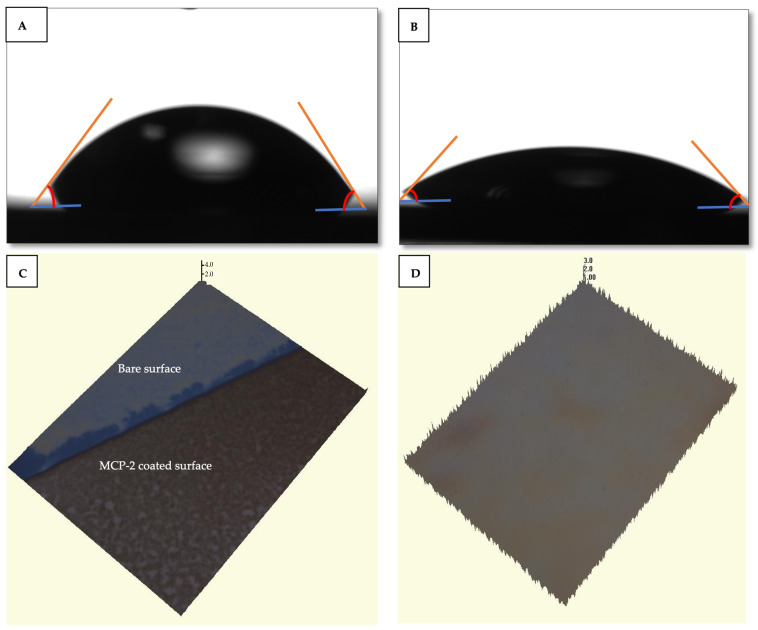
(**A**) Images of static contact angles of bare and (**B**) MCP-2-coated IRIS chips. (**C**) Optical profilometer images of bare and MCP-2-coated IRIS chips to show thickness of polymer-coated chip, and (**D**) optical profilometer image of MCP-2-coated IRIS chip.

The bare silicon surface exhibited a water contact angle of 85°, indicating a high level of hydrophobicity. Following the coating process, the water contact angle of the MCP-2 polymer-coated chip surface decreased to 52°. This alteration in the contact angle highlighted the modified SiO_2_ surface properties induced by the MCP-2 polymer. The gradual decrease in the water contact angle demonstrated the enhanced hydrophilicity of the surface, attributed to the successful coating of the surface with MCP-2 which can form a tri-dimensional polymer structure when in contact with water. The water contact angle images are shown in Figure 3A,B. The thickness of the MCP-2 polymer coating applied to the IRIS chip surface was verified to be 100 ± 1.1 nm. Optical profilometer images illustrating the measurements are provided in Figure 3C,D. These findings indicate the successful achievement of homogeneous polymer coating, confirming the effective performance of the MCP-2-coated IRIS chip. The MCP-2 polymer is an NHS-based reactive polymer, which allows for the elevation of anchored bioreceptors by forming a 3D structured monolayer film on biosensor chip surfaces. The free amino groups of MCP-2 make this polymer a good candidate for surface coating to immobilize proteins and amine-modified DNA, RNA or peptides via covalent binding in biosensor applications [21].

While examining the binding affinity of anti-VEGF drugs to their target, the main challenges for obtaining monovalent binding affinity for rhVEGF and its inhibitors are the dimeric form of rhVEGF and the presence of two binding sites on the inhibitors [8]. In this study, a low concentration of the analyte (rhVEGF, 1 nM) flowed across the bevacizumab and aflibercept spotted chip promoting monovalent binding.

After the injection of the sample to the IRIS cartridge, the accumulation of biomolecules on the spotted area depending on the interaction between anti-VEFG and rhVEGF occurred. Afterwards, the optical path difference resulting from biomolecule accumulation on the thin film surface of the sensor that was designed as a microarray was measured. The real-time characterization of VEGF binding was performed using IRIS-A and IRIS-B chips. First, PBS as an equilibration buffer flowed through the IRIS system at a 50 μL/min flow rate. Then, a 1 nM VEGF solution interacted across the surface of the chip in the IRIS system. The changes in the differential signal were monitored in real time.

**Figure 4:**
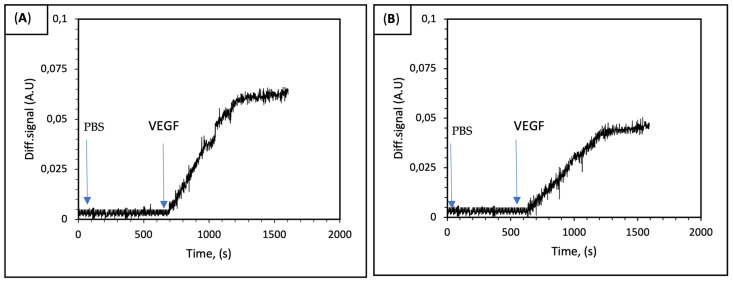
(**A**,**B**) The binding response of VEGF on IRIS-A and IRIS-B. The PBS buffer was initially run for the first 600 s, followed by the introduction of the 1 nM VEGF solution. Saturation was observed to occur approximately 1200 s after the beginning of the experiment.

Binding curves were obtained with the binding of rhVEGF to anti-VEGF drugs that spotted on the surface of the IRIS chip. On the other hand, a signal could not be recorded for the IgG and BSA spots used as the control. In Figure 4A,B signal enhancements of 0.06 and 0.04 were observed for IRIS-A and IRIS-B chips, respectively. The results demonstrated that both aflibercept and bevacizumab molecules exhibit nearly identical binding affinities to rhVEGF.

**Figure 5:**
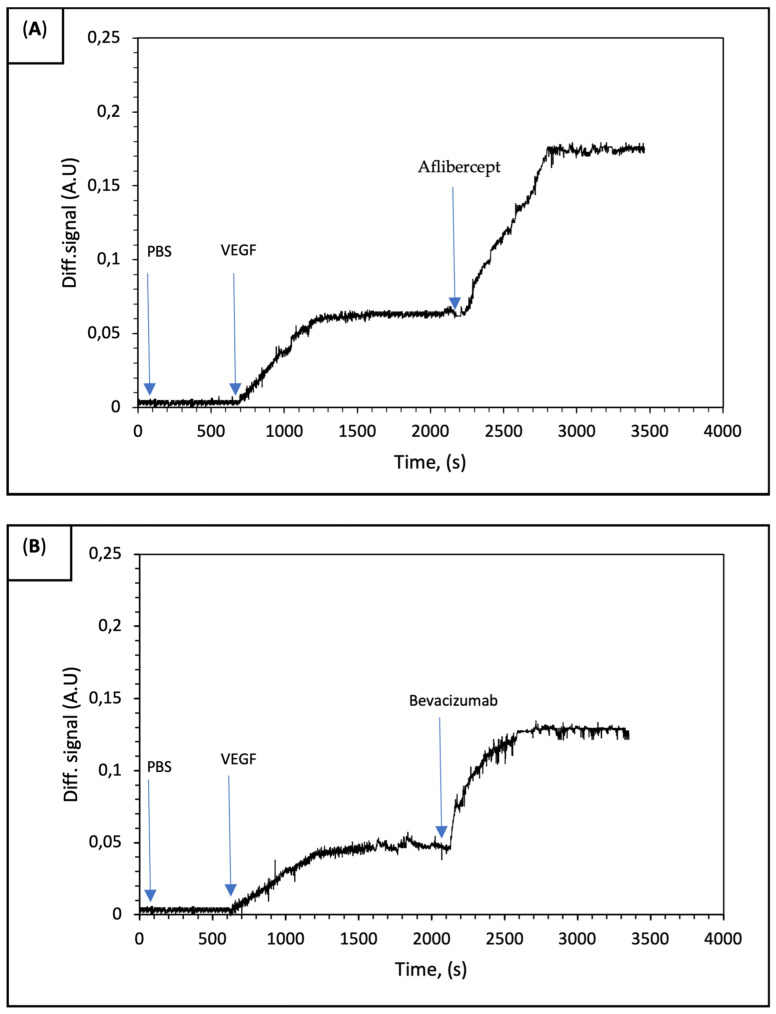
Showing sandwich assay model of binding VEGF and then anti-VEGF drugs: (**A**) First running VEGF and then aflibercept on IRIS-B spot and (**B**) running VEGF and then bevacizumab on IRIS-A. In both experiments, PBS buffer was initially run for first 600 s, then followed by 1 nM VEGF solution. Saturation occurred approximately 1200 s later. Aflibercept and bevacizumab were subsequently introduced at 2500 s.

With the use of the sandwich assay model, enhanced signal levels were recorded for each anti-VEGF drug (Figure 5). In the sandwich assay model used to study the binding of VEGF and anti-VEGF drugs, the PBS buffer was initially run for the first 600 s. This was followed by separately applying a 1 nM VEGF solution over each IRIS-A (Figure 5A) and IRIS-B spot (Figure 5B). Finally, to demonstrate the affinity of each molecule, aflibercept was applied to the IRIS-A spot and bevacizumab to the IRIS-B spot. The measurement data presented in Figure 4 are also shown in the initial section of Figure 5. This experiment demonstrates the binding affinity between VEGF–aflibercept and V–bevacizumab. The binding affinity distinctness of the two anti-VEGF drugs can be explained with the fundamental differences that were observed with their binding geometry. Unlike aflibercept, which constitutes a simple homogenous 1:1 molar binding complex with rhVEGF, bevacizumab forms large heterogenous complexes with rhVEGF [4]. Recent studies in the literature that measure VEGF concentrations after the intravitreal administration of anti-VEGF drugs have introduced that aflibercept is more efficient than bevacizumab to reduce circulating VEGF [22,23,24,25].

These data were confirmed with the in vitro assay based on the detection of VEGF by surface-captured antibodies for both in the presence of anti-VEGF and without anti-VEGF. When the assay was performed with bevacizumab, due to the lower binding affinity of this anti-VEGF drug, VEGF molecules dissociated from bevacizumab after sample incubation, and free VEGF was detected in the assay in contrast to aflibercept [7]. In a study of Papadopoulos et al. that investigated the binding affinity of anti-VEGF drugs to VEGF by SPR, aflibercept showed a higher binding affinity with a 0.49 pM KD value when compared with bevacizumab (KD: 58 pM) [26].

**Figure 6:**
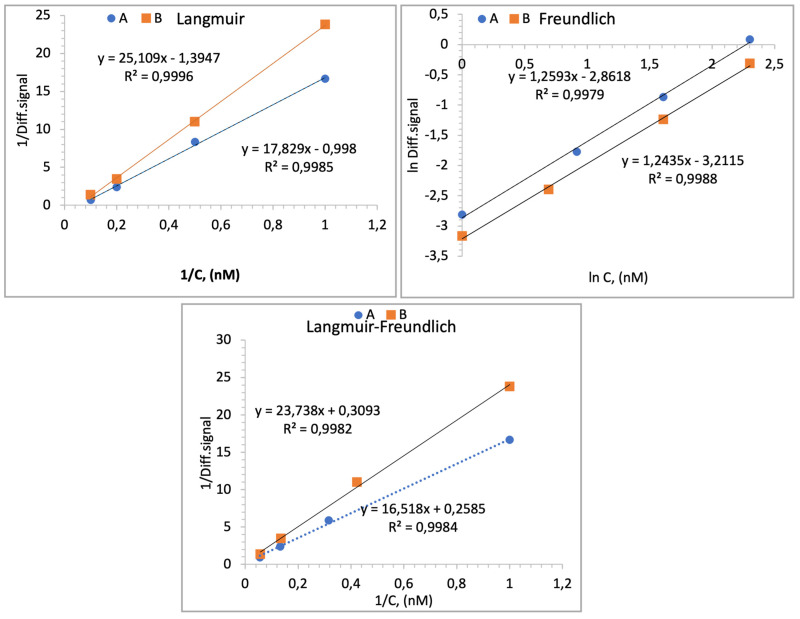
Isotherm models applied to data acquired on IRIS chips, A: aflibercept and B: bevacizumab.

The recorded data in this study using IRIS technology correlate with the data obtained with the SPR and ELISA methods in the literature [4,27,28]. Consequently, the findings suggest that the investigation of the binding affinity of AMD drugs to their target analytes (VEGF) has been successfully performed for the first time with an IRIS, which is a more cost-effective method than the SPR method. Isotherm parameters were obtained by fitting equilibrium data with the Langmuir, Freundlich and Langmuir–Freundlich isotherms for the adsorption. Also, these models are typically used to characterize binding in sequentially diluted solutions. It is assumed that the binding data obtained in this study were set with these isotherms when examined within average concentration ranges. The isotherm graphs are represented in Figure 6. The Langmuir isotherm assumes a monolayer surface, where the chip surface possesses an equal number of active sites and energy levels. Conversely, for heterogeneous surfaces, the Freundlich model, the most frequently utilized model, emerges as the initial derivation. The Langmuir–Freundlich adsorption isotherm model is determined as the most accurate for examining the binding behavior at elevated concentrations of both the VEGF–aflibercept and VEGF–bevacizumab molecules according to the following equations.

Langmuir isotherm:

ΔT = [ΔT_max_ C/K_D_ + C] (1)

Freundlich isotherm:

ΔT = [ΔT_max_ C 1/n] (2)

Langmuir-Freundlich isotherm:

ΔT = [ΔT_max_ C^1/n^/K_D_ + C^1/n^] (3)

ΔT_max_ is the maximum sensor signal change; ΔT is the equilibrium IRIS signal change; C is the concentration of VEGF (nM) in the solution; K_D_ (nM) is the dissociation constant; K_A_ (1/nM) is the association constant. Furthermore, the Freundlich (heterogeneity) exponent is defined as 1/n. Isotherm data of IRIS chips are given in Table 1.

The ΔT_max_ data can be applied to the Langmuir isotherm model, indicating that the sensor surface of the IRIS chip likely consists of a monolayer of probes, each possessing an equal number of binding sites per unit area.

The findings are significantly in agreement with the Langmuir model, suggesting that the surface of the IRIS chip consists of a homogeneously distributed monolayer with co-energy and minimal lateral interaction.

## 4. Conclusions

In conclusion, the limitations of existing detection technologies highlight the critical need for innovative and versatile sensing methods. This study has demonstrated the effectiveness of the Interferometric Reflectance Imaging Sensor (IRIS) in assessing the binding efficiencies of therapeutics, specifically for drugs used in the treatment of wet Age-related macular degeneration (AMD). Our findings affirm that the IRIS offers a cost-effective, highly sensitive and rapid alternative to traditional methods such as ELISA, which often rely on expensive chips and instruments. Looking ahead, IRIS technology shows great promise for further applications in biomedicine, including the determination of binding affinities for novel drugs and the comparison of various compounds in the development of new therapeutics.

## Figures and Tables

**Table 1:** Isotherm data of IRIS chips.

	Langmuir		Freundlich		Langmuir–Freundlich	
Aflibercept	T_max_ K_A_ K_D_ R^2^	1.02 1.05 17.46 0.998	T_max_ 1/n R^2^	2.35 1.25 0.997	T_max_ K_A_ K_D_ R^2^	4.01 0.15 66.0 0.998
Bevacizumab	T_max_ K_A_ K_D_ R^2^	0.71 0.2 3.5 0.999	T_max_ 1/n R^2^	2.31 1.24 0.998	T_max_ K_A_ K_D_ R^2^	0.04 1.009 0.99 0.998

## Data Availability

There are no data.
